# Acute Kidney Injury among Neonates with Perinatal Asphyxia in a Tertiary Care Centre

**DOI:** 10.31729/jnma.8402

**Published:** 2024-01-31

**Authors:** Arun Giri, Sunil Kumar Yadav, Vijay Kumar Shah

**Affiliations:** 1 Department of Paediatrics, Nobel Medical College Teaching Hospital, Biratnagar, Morang, Nepal

**Keywords:** *acute kidney injury*, *neonates*, *prevalence*

## Abstract

**Introduction:**

Perinatal asphyxia is a very common cause of morbidity and mortality in both term and preterm neonates and ranks the second most important cause of neonatal death. The incidence and importance of acute kidney injury in the neonatal period are becoming increasingly apparent. Early diagnosis and timely management of acute kidney injury is very important in these newborns to avoid other long-term adverse events. This study aimed to find out the prevalence of acute kidney injury among neonates with perinatal asphyxia in a tertiary care centre.

**Methods:**

A descriptive cross-sectional study was conducted at the neonatal intensive care unit and nursery in a tertiary care hospital of Eastern Nepal from 1 November 2021 to 31 October 2022 after obtaining ethical approval from the Institutional Review Committee. A convenience sampling method was used. The point estimate was calculated at a 95% Confidence Interval.

**Results:**

Among 97 neonates, the prevalence of acute kidney injury was 60 (61.86%) (50.25-69.75, 95% Confidence Interval). Out of which 42 (70%) were males and 18 (30%) were female.

**Conclusions:**

The prevalence of acute kidney injury among neonates with perinatal asphyxia was found to be higher than other studies done in a similar setting.

## INTRODUCTION

Perinatal asphyxia is a very common cause of morbidity and mortality in both term and preterm neonates and is the second leading cause of newborn mortality.^1^ The World Health Organisation (WHO) defines asphyxia as "the inability to initiate and sustain breathing at birth".^2^ Acute kidney injury (AKI) is more common in asphyxiated neonates, and this can adversely affect the overall prognosis.^3^

Acute kidney injury is a potentially fatal illness that affects over 13 million people annually, with 85% of those affected living in low- to middle-income nations.^4^ Among asphyxiated term neonates, the incidence of AKI is substantial (50-72%).^5^ Perinatal asphyxia is the third most common cause of newborn mortality in Nepal, as per WHO data published in 2018. With 1,000 live births, Nepal has a neonatal mortality rate (NMR) of 22.^6,7^

This study aimed to find out the prevalence of acute kidney injury among neonates with perinatal asphyxia in a tertiary care centre.

## METHODS

A descriptive cross-sectional study was conducted at the neonatal intensive care unit and nursery in a tertiary care hospital of Eastern Nepal from 1 November 2021 to 31 October 2022 after obtaining ethical approval from the Institutional Review Committee of the Nobel Medical College Teaching Hospital (NMCTH) (Reference number: IRC-NMCTH 548/2021). All term neonates admitted in NICU and Nursery from the labour room, operation theatre, post-natal ward, and emergency of (NMCTH) and all term (37-42 weeks of gestation) neonates born, APGAR (appearance, pulse, grimace, activity, and respiration) score of <7 at 5 minutes after birth, delayed cry of more than 5 minutes and need for positive pressure ventilation for more than 1 minute were included in the study. Neonates with known factors believed to alter renal function such as (septicemia, respiratory distress syndrome, necrotising enterocolitis, major congenital anomalies of the kidney and urinary tract), mother taking any medications that could affect the neonatal renal function test, neonates on IV nephrotoxic drugs were excluded from this study. The convenience sampling method was used. The sample size was calculated by using the following formula:


$$\begin{array}{ll} \text{n} & ={\text{Z}}^{2}\times \frac{\text{p}\times \text{q}}{{\text{e}}^{\text{2}}}\\ & =1.96^{2}\times \frac{0.56\times 0.44}{0.1^{2}}\\ & =95 \end{array}$$

Where,

n = minimum required sample size
Z = 1.96 at 95 % Confidence Interval (CI)
p = prevalence taken from the previous study, 56%^8^
q = 1-p
e = margin of error, 10%

The minimum sample size calculated was 95. However, we enrolled 97 patients. A full history and detailed examination of all the term asphyxiated neonates who fulfilled the inclusion criteria was done and findings of physical examination and systemic signs were recorded using a preformed proforma. On the basis of Apgar score at 5 minutes the asphyxiated babies were further grouped into mild (score of 6 or 7), moderate (score 5 or 4) and severe asphyxia (score 3 or less). All term neonates with clinical features of HIE were staged by the Sarnat and Sarnat scoring system.^9^

All enrolled babies were subjected to an ultrasonography within 24 hours of birth to rule out any congenital malformation of the urinary tract. Daily weight recordings were taken on an electronic scale, 24-hour urine output measurement was done by applying plastic collection bags and urine examination was performed every day. Renal function parameters- blood urea, serum creatinine, serum electrolytes, urinary sodium and creatinine were monitored initially within 24 hours of birth and then on day 3 of life. After 3 days those babies having abnormal renal functions had their laboratory parameters monitored every alternate day till recovery. Neonates who developed renal failure were managed conservatively as per the standard hospital protocol. Arterial blood gas analysis and ECG were done as and when required. Criteria adopted for labelling an asphyxiated neonate as having renal failure are urine output <0.5 mL/kg/hour, blood urea >40 mg/dL, serum creatinine >1 mg/dL, and the presence of significant hematuria or proteinuria. These criteria were applied on day 3 of life and any three of the four criteria when fulfilled were considered renal failure.^10^

The data was collected and entered in Microsoft Excel and analyzed using IBM SPSS Statistics version 22.0. The point estimate was calculated at a 95% CI.

## RESULTS

Among 97 neonates, the prevalence of acute kidney injury was seen among 60 (61.86%) (50.25-69.75, 95% Confidence Interval) neonates. The mean age in hours of the neonates was 7.00±7.55 hours. The mean duration of hospital stay was 5.66±3.85 days. Out of 63 neonates with AKI, 36 had APGAR scores between 6-7 (mild asphyxia) (Table 1).

**Table 1:** Severity of asphyxia (n= 60).

Garde of asphyxia	n (%)
Mild asphyxia	36 (60)
Moderate asphyxia	15 (25)
Severe asphyxia	9 (15)

**Figure 1:**
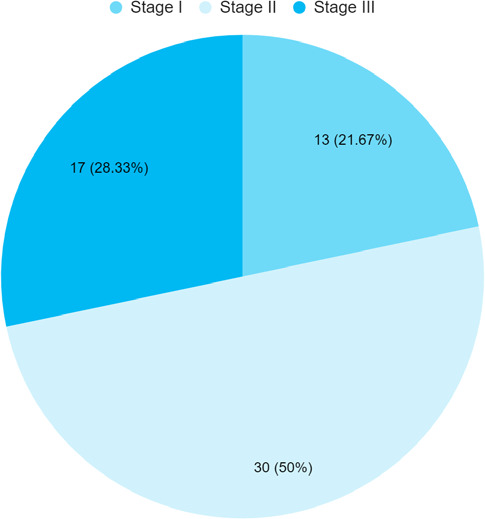
Stage of HIE among neonates with perinatal asphyxia with AKI (n= 60).

Out of 60 cases of perinatal asphyxia with AKI, 30 (50%) had stage II HIE (Figure 1).

Out of 60, 42 (70%) were males and 18 (30%) were female. The mean age of the mother was 24.37±3.36 years. The mean gestational week of pregnancy was 38.72±1.52 weeks. Among them, 28 (46.67%) neonates were born with cesarean section delivery, 27 (45%) neonates were born via vaginal delivery and 5 (8.33%) neonates were born via vacuum-assisted delivery. Among 60 neonates with AKI, 44 (73.33%) neonates were born to primigravida mothers and 16 (26.67%) neonates from multigravida mothers. A total of 26 (43.33%) were born through meconium-stained liquor. The mean serum Cr was 1. 1021±0.41 mg/dl. The mean serum urea was 39.26±16.12 mg/dl. The mean serum sodium was 133.86±4.76 mEq/l. The mean serum potassium was 4.63±0.60 mEq/l. The mean haemoglobin (Hb) was 17.14±2.31 mg/dl. The mean TLC was 15070±5.27 /mm^3^. The mean platelet was 274,000± 9.64/mm^3^.

A total of 52 (86.67%) neonates had oliguric acute kidney injury and in hospital mortality was seen in 7 (11.67%).

## DISCUSSION

In our study, among 97 neonates, the prevalence of acute kidney injury was seen among 60 (61.86%) which is higher as compared to other studies done in Nepal.^11,12^ In our study, 42 (70%) were males indicative of male preponderance and there were other studies in Nepal done with similar findings.^11,12^ This discrepancy could be explained by the fact that our hospital is a tertiary care hospital and is a referral hospital for many district and zonal hospitals with a capacity of 40 NICU beds and 6 Nursery beds.

In our study, stage II HIE is the commonest which is similar to the finding of a study from India done where the majority of cases were in HIE stage II accounting for 45.71%.^13^ Similarly, the occurrence of AKI was most commonly seen with HIE stage II neonates and a similar finding was noted from a study done in Nepal.^11,14^ Among 60 neonates who developed AKI, 28 (46.67%) neonates were born with cesarean section delivery, 27 (45%) neonates were born via vaginal delivery and 5 (8.33%) neonates were born via vacuum-assisted delivery in contrast to the finding from one of the studies done in India which showed that 56.7% of the asphyxiated babies were delivered by spontaneous vaginal delivery, 16.5% by assisted vaginal delivery and C/S in 27.1%.^15^ In our study the mean gestational week of pregnancy was 38.72±1.519 weeks. The mean gestational age of our study is similar to a cross-sectional study conducted in India.^14^ The previous study showed that the mean urine output (UOP) was 1.9±0.6 ml/kg/hr. This revealed that most of our AKI patients were non-oliguric, where oliguria is defined as UOP <1 mL/kg/h.^16^ Out of 60 asphyxiated neonates with AKI, 52 (86.67%) patients had oliguric acute kidney injury and these findings were similar to findings from one study done in India.^3^

There are a few limitations in our study. Although the study involved a diverse sample of critically sick neonates, it was conducted at a single centre, raising concerns about the generalizability of the results to other healthcare settings.

## CONCLUSIONS

The prevalence of acute kidney injury among neonates with perinatal asphyxia was found to be higher than other studies done in a similar setting.
